# Function of Mouse Embryonic Stem Cell-Derived Supporting Cells in Neural Progenitor Cell Maturation and Long Term Cxpansion

**DOI:** 10.1371/journal.pone.0054332

**Published:** 2013-01-14

**Authors:** Yunqian Guan, Qing-An Du, Wanwan Zhu, Chunlin Zou, Di Wu, Ling Chen, Yu Alex Zhang

**Affiliations:** 1 Cell Therapy Center, Beijing Institute of Geriatrics, Xuanwu Hospital, Capital Medical University, Beijing, P. R. China; 2 Department of Neurosurgery, Xuanwu Hospital, Capital Medical University, Beijing, P. R. China; 3 Key Laboratory of Neurodegeneration, Ministry of Education, P. R. China; Instituto Butantan, Brazil

## Abstract

**Background:**

In the differentiation of mouse embryonic stem (ES) cells into neurons using the 5-stage method, cells in stage 4 are in general used as neural progenitors (NPs) because of their ability to give rise to neurons. The choice of stage 4 raises several questions about neural progenitors such as the type of cell types that are specifically considered to be neural progenitors, the exact time when these progenitors become capable of neurogenesis and whether neurogenesis is an independent and autonomous process or the result of an interaction between NP cells and the surrounding cells.

**Methodology/Principal Findings:**

In this study, we found that the confluent monolayer cells and neural sphere like cell clusters both appeared in the culture of the first 14 days and the subsequent 6 weeks. However, only the sphere cells are neural progenitors that give rise to neurons and astrocytes. The NP cells require 14 days to mature into neural lineages fully capable of differentiation. We also found that although the confluent monolayer cells do not undergo neurogenesis, they play a crucial role in the growth, differentiation, and apoptosis of the sphere cells, during the first 14 days and long term culture, by secreted factors and direct cell to cell contact.

**Conclusions/Significance:**

The sphere cells in stage 4 are more committed to developing into neural progenitors than monolayer cells. Interaction between the monolayer cells and sphere cells is important in the development of stage 4 cell characteristics.

## Introduction

Mouse embryonic stem cells (ES) have the potential to differentiate into many cell types and are thus considered potential cell therapy candidates to treat neurodegenerative diseases [1]–[3].To avoid teratoma formation in ES cells and prevent damage to fully differentiated mature neurons during transplantation, ES derived neuronal progenitor cells (NPC) are the preferred cell types in neural degenerative disease research [4]–[7]. Understanding the development of neural progenitor cells becomes important.

In mouse, the most frequently used technique to differentiate ES cells to neurons is the 5-step method [8]–[10], and stromal-derived inducing activity (SDIA) method. In 5-step method, cells in the expanding stage (stage 4) are used as NPCs [6], [11]–[13]. Given SDIA method, ES cells cultured on PA6 or MS5 feeder cells for a specific period are also used as NPCs [14]–[18]. In both of the methods, the developmental process of neural progenitors in vitro also remains to be addressed. First of all, what cell type is more committed neural progenitor? Or in another word, the critical time when the neural-progenitors are fully competent to undergo neurogenesis and the time of their isolation from other surrounding cells that are not undergoing neurogenesis are yet to be determined. Can these more committed neural progenitors be passaged without losing their potential to differentiate into neurons? The fate and function of cells that do not undergo neurogenesis is yet another interesting question to be answered. Are these cells helpful in the differentiation of NPCs into neurons or are they byproducts of the differentiation?

Cumulating evidences suggest that NPCs can be expanded. Human ES cell derived NPCs maintain the ability to undergo neurogenesis during a long term culture [19]. Chung et al [20] isolated Otx2^+^ Corin^+^ NP cells at the end of stage 3 and maintained them for 4 weeks with 1,000-fold expansion without significant changes in their phenotype. Similarly, Hayashi et al obtained “adherent neurospheres” with a modified EB formation method and cultured them for 12 weeks [21]. All these results suggest that the NP cells could be cultured for longer duration and harvested in higher quantities.

Other evidences suggest some cells in NPC are more committed to neurons, and the neurogenesis of mES derived neural progenitors is not an autonomous process, but is influenced by surrounding cells. For example, the critical role of the in vitro or in vivo microenvironment in the differentiation of stem cells or NPCs has been studied. Transplantation of the ES cells cultured on MS5 or PA6 for longer time could increase the neural differentiation of graft and decrease the potential of tumor formation risk significantly [15], [22]–[24]. PA6 cell surface activity is required for neural differentiation of hESC, but secreted factors are required for the specific DA neuron-inducing effect [25]. Transplantation of stem cells into different areas of the brain results in a difference in differentiation suggesting that the fate of the graft is influenced by cell-cell contact and secreted factors released either by the graft or the host [26]–[28].

In our previous report [29], we observed two cell types on day 14 in the mouse ES cell derived neural progenitor expanding stage, which is stage 4. We reported that one cell type grows like neural spheres and are scattered among the second cell type, which are the confluent monolayer of cells. While the neural sphere cells give rise to neurons and astrocytes, the confluent monolayer cells do not. But we found that the monolayer cells support the growth of spheres by inducing their proliferation, decreasing apoptosis and increasing the overall percentage of neuron formation.

In this study, we divided the 8 week long stage 4 into the first 14 days and the subsequent 6 weeks, and explored whether stage 4 cells could be divided into spheres and monolayer cells during the two periods, and more importantly, whether sphere cells had the potential to be neural progenitors. Because NPCs in general require some time to obtain the complete ability to give rise to neurons, we also determined the number of days required for the mouse ES derived NPCs to obtain the ability to undergo neurogenesis. In addition, we studied the effects of monolayer cells on sphere cell differentiation into neurons during the first 14 days and the next 6 weeks, and found that it was achieved by cell-cell contact and (or) secreted factors.

## Results

### 1. Sphere Cells and Monolayer Cells Exist in the First 14 Days and the Subsequent 6 Week Culture of Stage 4 Cells

**Figure 1:**
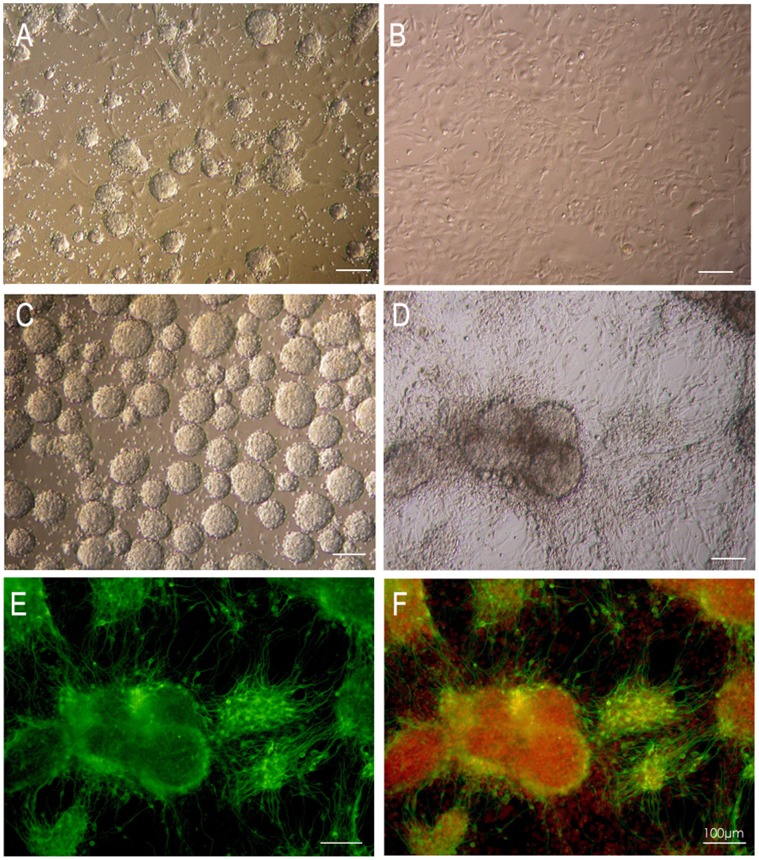
Typical cell types of stage 4 are sphere cells and monolayer cells. (A) Cell aggregates and surrounding monolayer cells at the beginning of stage 4. Within 14 days, the spheres will grow bigger and the monolayer cells will reach 90% confluence. (B)The independently cultured monolayer cells after the removal of cell aggregates by collagenase IV. (C) After collagenase treatment and sorting with 40 µm mesh, the cell aggregates can be disassociated from the surrounding cells and cultured in suspension to form spheres. (D) Phase contrast image of stage 5 cells finished differentiation. (E)Tuj-1staining of the stage 5 cells after 7 days of differentiation. The neurons with Tuj-1 staining are located mainly in the cell clusters. Tuj-1 positive cells could be hardly found in monolayer cells. (F) Merged image of the Tuj-1 staining and DAPI staining of nucleus in the same field. Green, Tuj-1; Red, DAPI. Scale bars, 100 µm.

The cell morphology during stage 4 is characterized by cell aggregates scattered amongst monolayer cells in addition to numerous fibers between the ell aggregates (Fig. 1A). In our experiments, when cells in stage 3 are disassociated and plated into stage 4, cell aggregates form on day two or three after plating and can be separated from the monolayer using collagenase. Morphology of these detached cell aggregates are similar to neurospheres isolated from fetal or adult nerve tissue of rodents or humans. Because of this similarity we coin the term “sphere cells” in this study. In contrast, cells surrounding the cell aggregates are termed as “monolayer cells” due to their characteristic morphology.

In general 5–7 days after plating, stage 4 cells attain 80% confluence and are ready for passage. Repeated passing and 8 weeks of continuous culture resulted in monolayer (Fig. 1B) and sphere cells (Fig. 1C) both of which can be separated and cultured in the respective stage 4 medium.

We also found that when stage 4 cells start differentiation to stage 5 on day 14, the percentage of Tuj-1 positive neurons (Fig. 1D, E, F) is 60–70% and GFAP positive astrocytes is 7–12%. In addition, during the subsequent 6 weeks of culture, cells differentiate into stage 5 and on week 5 and 8 have similar percentages of neurons and astrocytes. This indicates that the ability of stage 4 cells to undergo neurogenesis remains unchanged during long culture periods.

### 2. Sphere Cells are Neural Progenitor Cells during the First 14 Days and the Subsequent 6 Weeks of Stage 4

**Figure 2:**
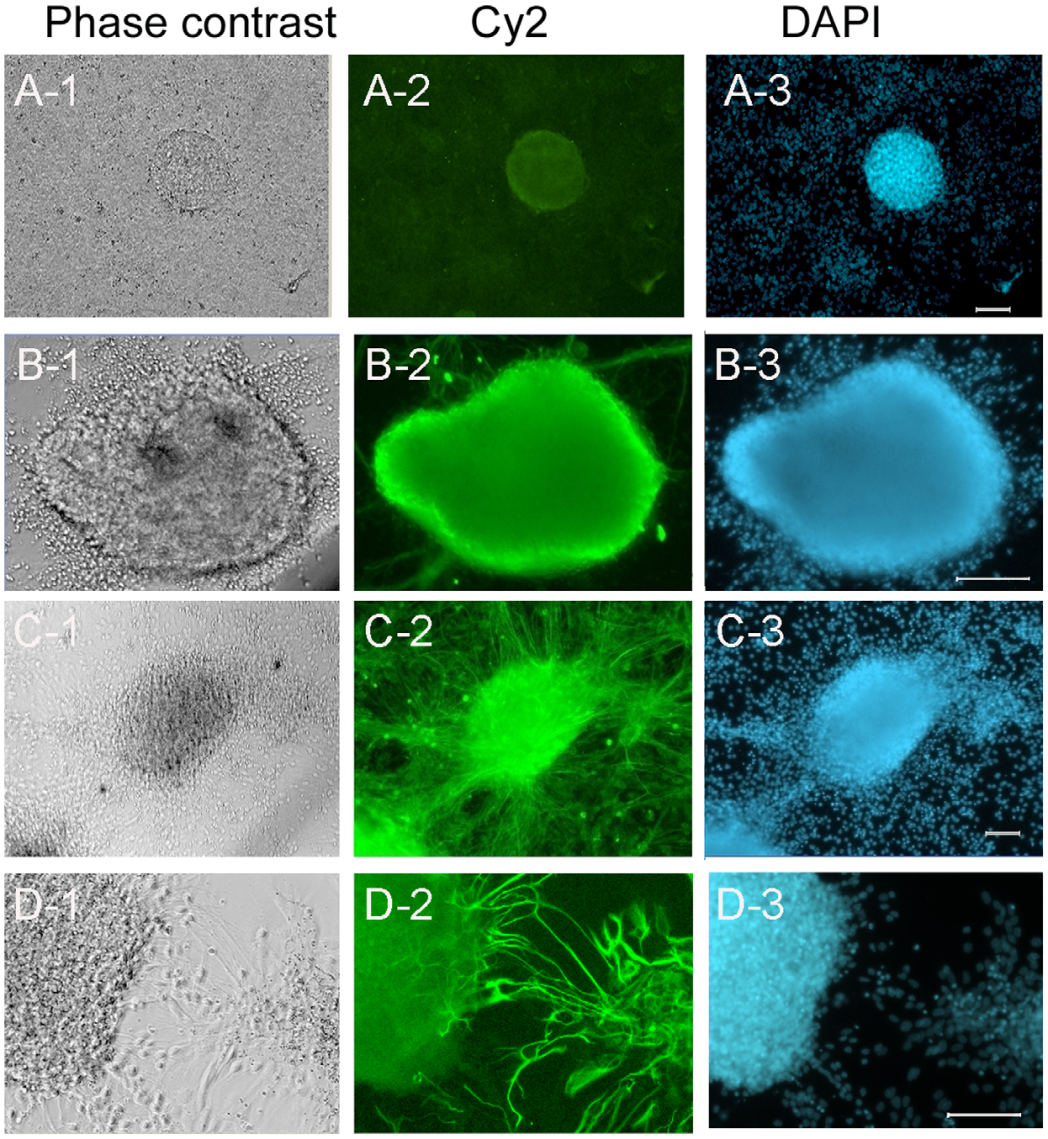
Characterization of sphere cells. (A) The sphere cells isolated by the end of the long term culture in stage 4 still contain Oct-4 positive cells. (B) The sphere cells are nestin positive. (C) After 5 stages of differentiation, the sphere cells alone gave rise to 35–40% Tuj-1positive neurons. (D) After 5 stages of differentiation, GFAP positive astrocytes derived from sphere cells were 6–9%. Green in A-2, B-2, C-2 and D-1 are Oct-4, nestin, Tuj-1, and GFAP. Blue, DAPI. Scale bars, 100 µm.

Spheres are oval or round with diameters of 100–300 nm and are not completely differentiated even after stage 5. While most sphere cells are nestin positive (Fig. 2B1–B3), undifferentiated Oct-4 positive cells do exist (Fig. 2A1–A3).

We found that spheres are mainly composed of neural progenitors. Stage 4 sphere cells were separated on day 14, cultured for subsequent 6 weeks, and differentiated independently at week 5 and 8 in stage 5 medium for 7 days. The numbers of neurons and astrocytes were counted after Tuj-1 and GFAP staining. Without monolayer cells, the spheres give rise to 35–40%Tuj-1 positive (Fig. 2C1–C3) neurons and 6–9% GFAP positive astrocytes (Fig. 2D1–D3).

**Figure 3:**
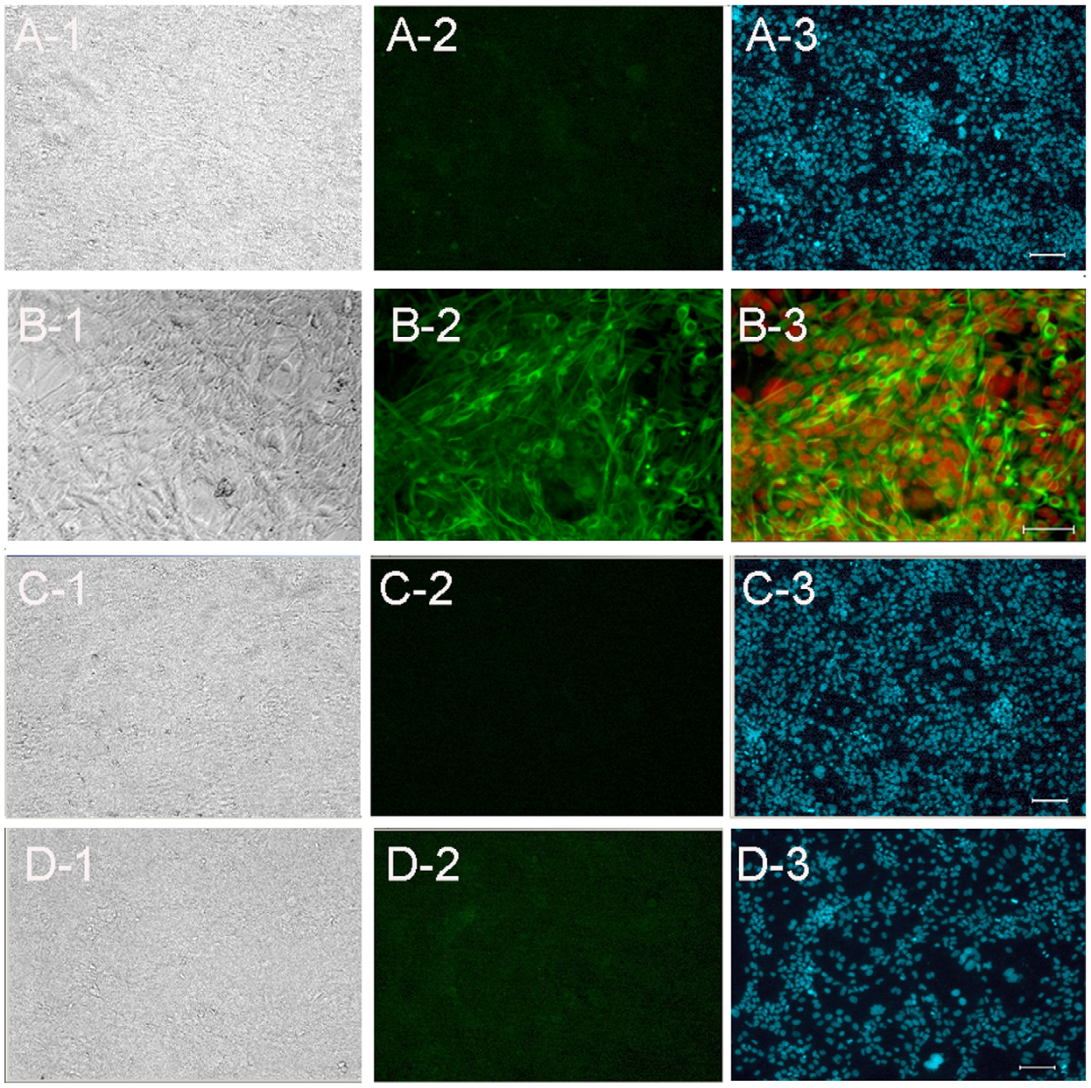
Characterization of confluent monolayer cells. (A) At the end of 8 weeks long culture of stage 4, the separated monolayer cells are negative for Oct-4. (B) The flat monolayer cells are positive for nestin. (C) The flat monolayer cells are negative for neuron marker, Tuj-1, and negative for astrocyte marker GFAP (D). Green, nestin; Red, DAPI; Blue, DAPI. Scale bars, 100 µm (A, C and D) and 50 µm (B).

However, throughout the 8 week culture of stage 4, monolayer cells lack Oct-4 positive cells (Fig. 3A1–A3), but are positive for nestin (Fig. 3B1–B3). Regardless of the time of testing, these monolayer cells are neither progenitors of neurons (Fig. 3C1–C3) nor astrocytes (Fig. 3D1–D3).

### 3. First 14 Days in Stage 4 is Essential for NPC to Obtain Continuous Ability for Neurogenesis

In the experiments about stage 4 cells as a whole and sphere cells alone. To avoid the impact of collagenase disassociation on the characteristics of sphere cells alone, stage 4 cells used in these experiments were recombined sphere cells and monolayer cells. In brief, sphere cells were separated by collagenase, then plated on the monolayer cells, and differentiated in stage 5 medium.

**Figure 4:**
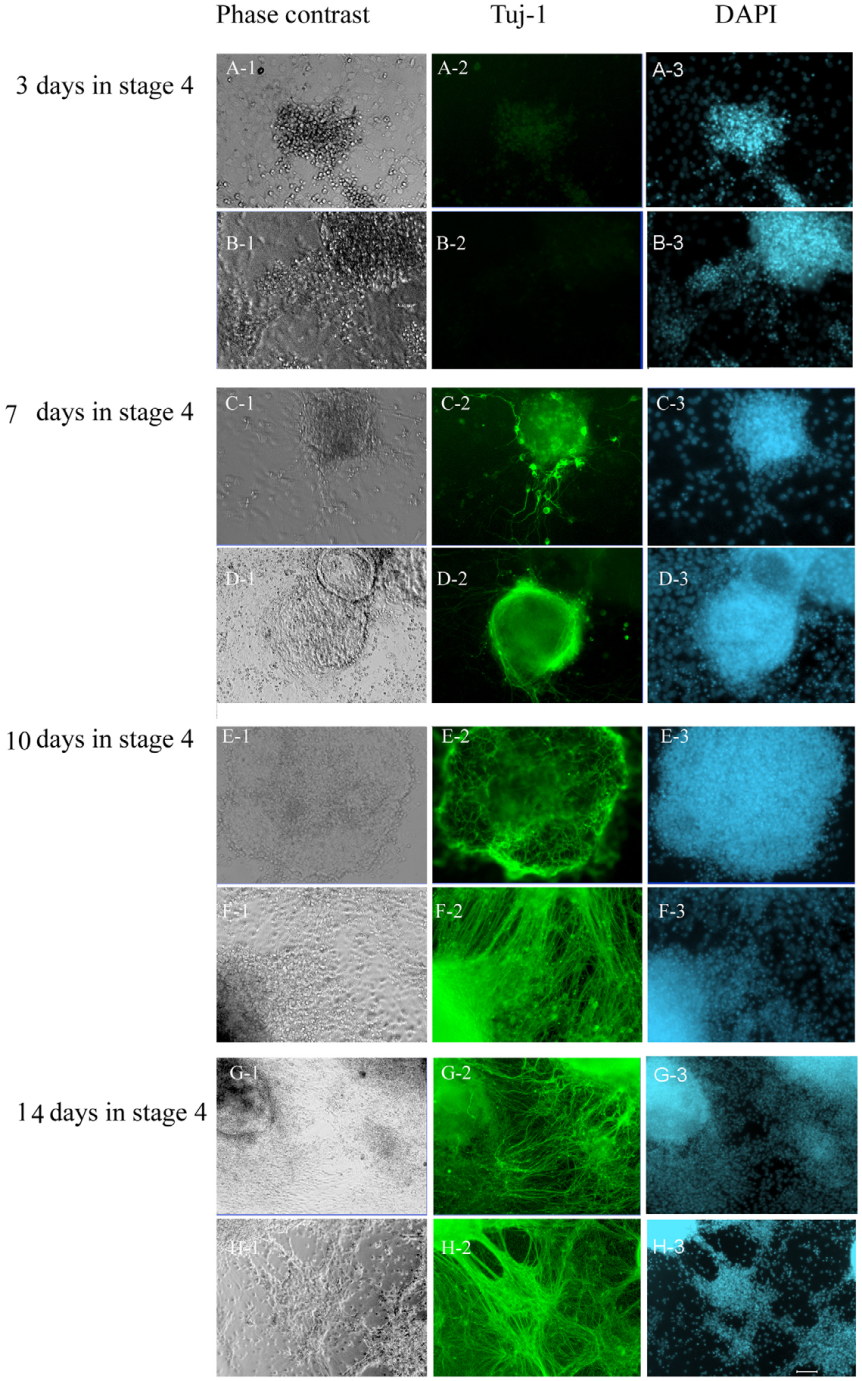
Two weeks in stage 4 is necessary for sphere cells to obtain complete ability to differentiate into neuron. The sphere cells (A, C, E, G) on days 3, 7, 10 and 14 in stage 4, were separated from monolayer cells and then differentiated alone or combined with monolayer cells. The Tuj-1 positive neurons in spheres isolated on day 3(A), 7(C), 10(E) and 14(G) reached the percentages of 0, 12.3±5.2, 22.4±7.9, and 39.9±5.7%. Each time point was significantly higher than the previous (p<0.05). In the presence of monolayer cells, the spheres isolated on day 3(B), 7(D), 10(F), and 14(H) in stage 4 gave rise to increasing numbers of neurons with percentages of 0, 17.3±6.4, 48.1±3.0, and 68.6±11.3%. The later time points were significantly higher than the previous. Green,Tuj-1; Blue, DAPI. Scale bars, 50 µm.

To determine the time required for stage 4 cells to obtain the ability to undergo complete neurogenesis, we isolated sphere cells on days 3, 7, 10, and 14 in stage 4 and started stage 5 differentiation. The percentage of Tuj-1 positive neurons after stage 5 differentiation was significantly higher in later than earlier days, and was 0, 12.2±5.2, 22.4±7.9, and 39.9±5.7% (p<0.05) (Fig. 4A, 4C, 4E, 4G).

The re-combined sphere cells and monolayer cells also started to differentiate on days 3, 7, 10, and 14 in stage 4,and the percentage of neurons from these cells significantly increased with the increase in the number of days in culture, from zero to 17.3±6.4, 48.1±3.0, and 68.6±11.3% (p<0.05) (Fig. 4B, 4D, 4F, 4H). These results demonstrate higher neural yields than sphere cells alone in each time point. This test in addition also served to observe the effect of monolayer cells on the neurogenesis of sphere cells.

During the subsequent 6 weeks in culture, stage 4 cells did not require another 14 days in stage 4 medium to obtain the complete potential to differentiate into neurons. Usually they need 5–7 days to reach 80% confluence, and when transferred into stage 5 medium for differentiation, as high as 60–70% neurons were observed. Sphere cells alone, meanwhile, yielded only 40–45% when grown in the subsequent 6 weeks.

### 4. The Effect of Monolayer Cells on the Proliferation of Sphere Cells

Whether proliferation, apoptosis and differentiation characteristics of ES derived NPCs are autonomous or influenced by neighboring cells are not clear yet.

**Figure 5:**
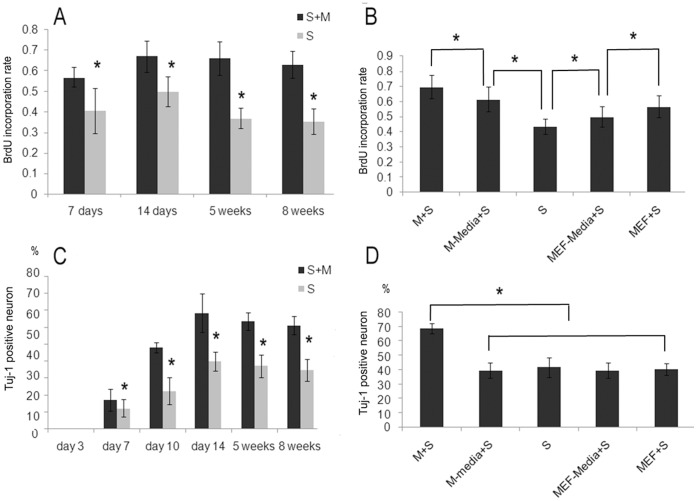
The effect of monolayer cells on the proliferation and differentiation of sphere cells. (A) At the time point of day 7 and 14, week 5 and 8 in stage 4, the BrdU incorporation rate of spheres alone was significantly lower than in the combined sphere cells and monolayer cells. (B) MEFs had a similar effect on sphere cell proliferation as monolayer cells at week 5. The BrdU incorporation rate of sphere cells cultured in conditioned medium of both monolayer cells and MEFs were significantly higher than sphere cells cultured in fresh stage 4 medium, but was not as high as that in combined monolayer and sphere cells. This indicates that secreted cytokines contribute to the proliferation of sphere cells mostly. (C) During the first 14 days and the next 6 weeks of stage 4, the percentage of neurons derived from sphere cells alone was always significantly lower than that in combined sphere and monolayer cells. This suggests that differentiation of neural progenitors into neurons is prompted by monolayer cells. (D) MEFs did not prompt the differentiation of sphere cells. The sphere cells cultured with the conditioned medium from monolayer cells or MEFs gave rise to similar percentages of neurons as sphere cells alone in fresh medium, but still dramatically lower than the sphere cells on monolayer cells (* p<0.05, n = 4–5). (Abbreviations: MEF: mouse embryonic fibroblast, M: monolayer cells; S: sphere cells; M-media: monolayer cell conditioned medium; MEF-media: MEF conditioned medium).

BrdU incorporation rate of stage 4 sphere cells on days 7 and 14 in culture are 40.7±11.0 and 50.0±7.4% and on weeks 5 and 8 are 43.3±5.0 and 42.3±6.3% respectively. These values are significantly lower than BrdU incorporation rates observed when sphere and monolayer cells are cultured together, which are 57.0±4.8, 67.1±7.6, 66.0±8.1 and 63.00±6.6% respectively. This suggests that proliferation of sphere cells in vivo are not autonomous but rather requires supporting cells, monolayer cells in our experiment, for growth and maximum proliferation (Fig. 5A).

In the next step, we tested if the proliferation effect of monolayer cells can be replaced by other cells. For this, week 5 in stage 4 was selected as the time point and CF1 mouse embryonic fibroblasts (MEFs), which is a commonly used feeder cell in ES culture, was used as substitution for monolayer cells. We found that MEFs also significantly increased the rate of BrdU incorporation (56.8±7.2%) in sphere cells. This showed that cells such as MEFs also promote sphere cell proliferation albeit not as much as monolayer cells. Thus, the ability to promote sphere cell growth is not unique to monolayer cells.

We further studied whether the effect of monolayer cells or MEFs on sphere cell proliferation is a result of cell-cell contact or secreted cytokines. The BrdU incorporation rate in sphere cells cultured in medium conditioned with both monolayer cells and/or MEFs is significantly higher than in fresh stage 4 medium. This implies that secreted cytokines contribute to the proliferation of sphere cells. However, the BrdU incorporation rate in sphere cells cultured in the monolayer or MEF conditioned medium was not as high as that cultured with monolayer cells. This clearly indicates that cell-cell contact is also involved in sphere cell growth (Fig. 5B).

### 5. The Effect of Monolayer Cells on the Differentiation of Sphere Cells

To determine the effect of monolayer cells on sphere cell differentiation, sphere colonies were isolated as described above and differentiated in stage 5 medium. Sphere cells in combined cultures (spheres and monolayer cell) also underwent a similar process of differentiation.

During the first 14 days in stage 4, we picked sphere cells on days 3, 7, 10 and 14 for differentiation to stage 5 with or without monolayer cells. Percentages of Tuj-1 positive neurons from sphere cells alone were 0, 12.3±5.2, 22.4±7.9 and 39.9±5.7% respectively. However, in the combined cultures with monolayer cells, the percentage of neurons in sphere cells was significantly (p<0.05) higher in the last three time points (17.3±6.4, 48.1±3.0, and 68.6±11.3%) (Fig. 5C).

The percentages of GFAP positive astrocytes in sphere cells that began to differentiate on days 3, 7, 10 and 14 in stage 4 without monolayer cells were 0, 7.3±3.7, 5.3±3.3, and 2.7±1.3% respectively. When monolayer cells were included in the culture, the sphere cells differentiated into 0, 12.8±5.2, 10.9±3.4 and 7.7±1.4% of astrocytes. In the last three time points, astrocyte percentage in the presence of monolayer cells are significantly higher than in their absence (p<0.05) (Fig. S1).

In the following long term culture of stage 4, the Tuj-1 positive neurons after differentiation of spheres alone on weeks 5 and 8 were 37.3±6.7 and 34.8±6.3%, which was lower when compared to 63.6±5.1 and 61.2±5.4% when combined with monolayer cells (Fig. 5C).

We also tested whether the ability of monolayer cells to promote differentiation could be replaced by MEFs, during long term culture of stage 4 cells. When co-cultured with MEFs, sphere cells gave rise to only 40–45% Tuj-1 positive neurons and 4.5–5% GFAP positive astrocytes which are similar to the percentages of differentiation with sphere cells alone. This showed that MEFs do not have the ability to promote differentiation of neurons or astrocytes as monolayer cells. In other words, monolayer cells have the unique potential to promote sphere cell differentiation into neurons.

To understand the role of cell-cell contact and secreted factors of sphere and monolayer cells in neurogenesis, we compared the neuron percentage after differentiation using a conditioned medium method.

The results showed that the percentage of neurons was 68.6±3.5% in stage 4 cells that includes normally formed sphere and monolayer cells. In the group with sphere cells cultured with conditioned medium from monolayer cells, the percentage of neurons was significantly lower (39.6±5.4%) and was the similar (41.8±6.7%) when sphere cells differentiated alone without any supporting cells. Culture of sphere cells together with MEF or in MEF conditioned medium didn’t increase the percentage of neurons either, which were 40.5±3.8% and 39.6±5.4% (Fig. 5D).

### 6. The Effect of Monolayer Cells on Apoptosis of Sphere Cells

**Figure 6:**
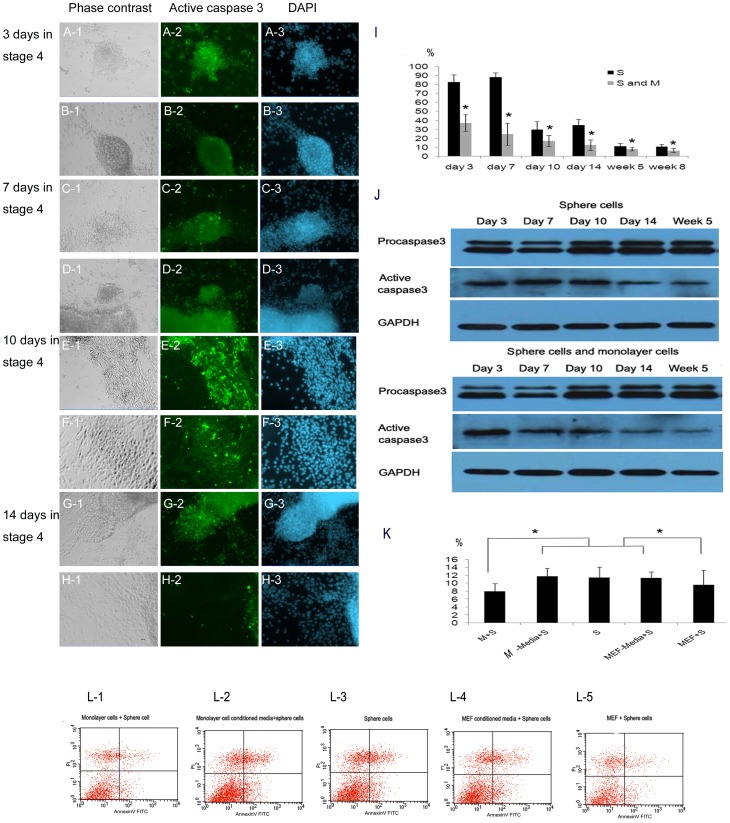
The effect of monolayer cells on the apoptosis of sphere cells. The sphere cells were separated from monolayer cells on days 3, 7, 10 and 14 in stage 4 and then were cultured alone or combined with monolayer cells for another 2 days before active caspase3 staining. (A), (C), (E), (G) are sphere cells alone, (B), (D), (F), (H) are combined sphere and monolayer cells. (I) During the first 14 days and the next 6 weeks of continuous culture and passage the sphere cells were isolated on days 3, 7, 10 and 14 and, weeks 5 and 8. At all time points, the apoptosis of sphere cells alone was significantly higher than that together with monolayer cells. (J) Analysis for procaspase-3 cleavage and active caspase 3 by immuneblotting confirmed these tendencies. (K) On week 5 in stage 4, MEFs had a similar effect as monolayer cells in preventing sphere cells from apoptosis. Therefore, the anti-apoptotic effect is not unique to monolayer cells. The apoptosis rate of sphere cells cultured in the conditioned medium from monolayer cells or fibroblasts, are 11.79±1.99 and 11.34±1.57%, which are not different from the apoptosis rate of sphere cells cultured in fresh medium (* p<0.05, n = 5). (L) Besides cell counting, the percentages of apoptotic (Annexin V-positive, PI negative) cells in sphere cells cultured in the conditioned medium from monolayer cells (10.1±0.8%) and from fibroblasts (11.0±0.7%) are higher than that in sphere cells cultured together with monolayer cells (7.8±0.8%) or fibroblasts (8.1±0.7%) (p<0.05, n = 5), but not differ from the apoptosis rates of sphere cells cultured in fresh medium (11.1±0.2%). (Abbreviations: MEF: mouse embryonic fibroblast, M: monolayer cells; S: sphere cells; M-media: monolayer cell conditioned medium; MEF-media: MEF conditioned medium). Green, active caspase 3; Blue, DAPI. Scale bar, 20 µm.

To detect apoptosis, active caspase 3 antibody staining was used. During the first 14 days in stage 4 culture, the apoptosis rates of sphere cells isolated on days 3, 7, 10 and 14 and expanded in stage 4 medium for another 2 days without monolayer cells were 82.8±7.8, 88.4±4.6, 30.0±8.7 and 34.7±6.6%. When sphere cells and monolayer cells were separated on days 3, 7, 10, 14 followed by culturing as a whole for 2 days, apoptosis rates of 37.2±9.4, 24.8±12.3, 17.1±6.1, and 12.5±5.8% (Fig. 6A–6H) were achieved(Fig. 6I). During the next 6 weeks of continuous culture and passing, the apoptosis rate of combined sphere cells and monolayer cells was 8.0±1.9 and 6.5±2.3% on weeks 5 and 8. At the same time points in stage 4, the apoptosis rate of sphere cells which were cultured without monolayer cells for 2 days, increased to 11.5±2.6 and 10.9±2.7% (Fig. 6I). At all time points, apoptosis was significantly higher with sphere cells alone compared to that when sphere and monolayer cells were combined. These results and the decreasing apoptosis tendency of sphere cells isolated on days 3, 7, 10 and 14 were confirmed by western blot using active caspase-3 antibody (Fig. 6J).

We also examined whether the anti-apoptotic effect is achieved by cell-cell contact or secreted factors. Apoptosis rate of sphere cells cultured in the conditioned medium from either monolayer cells (11.8±2.0%) or from MEF (11.3±1.6%) are not different from the apoptosis rates of sphere cells cultured in fresh medium(11.5±2.6%), but higher than that in sphere cells cultured together with monolayer cells (8.0±1.8%)or MEF(9.6±3.7%) (Fig. 6K). This highlights that it is more important of cell-cell contact than secreted factors in the anti-apoptotic effect. The fact that the effects of MEF in preventing sphere cells from undergoing apoptosis is similar to the monolayer cells suggest that the anti-apoptotic effect is not unique to monolayer cells. All these results suggest that without monolayer cells, the sphere cells were more vulnerable to apoptosis. The results were also confirmed by annexin V and propidium iodide (PI) flow cytometry detection (Fig. 6L).

### 7. Interaction of Monolayer and Sphere Cells Leads to Tumor Formation

A critical issue to be considered in cell transplantation therapy is the risk of teratoma formation. The ability of pre-differentiated ES cells to form teratoma remains unclear and conflicting results have been reported so far. Because Oct-4 staining is observed in sphere cells, we previously hypothesized that sphere cells may be the cause of tumor formation after transplantation.

**Figure 7:**
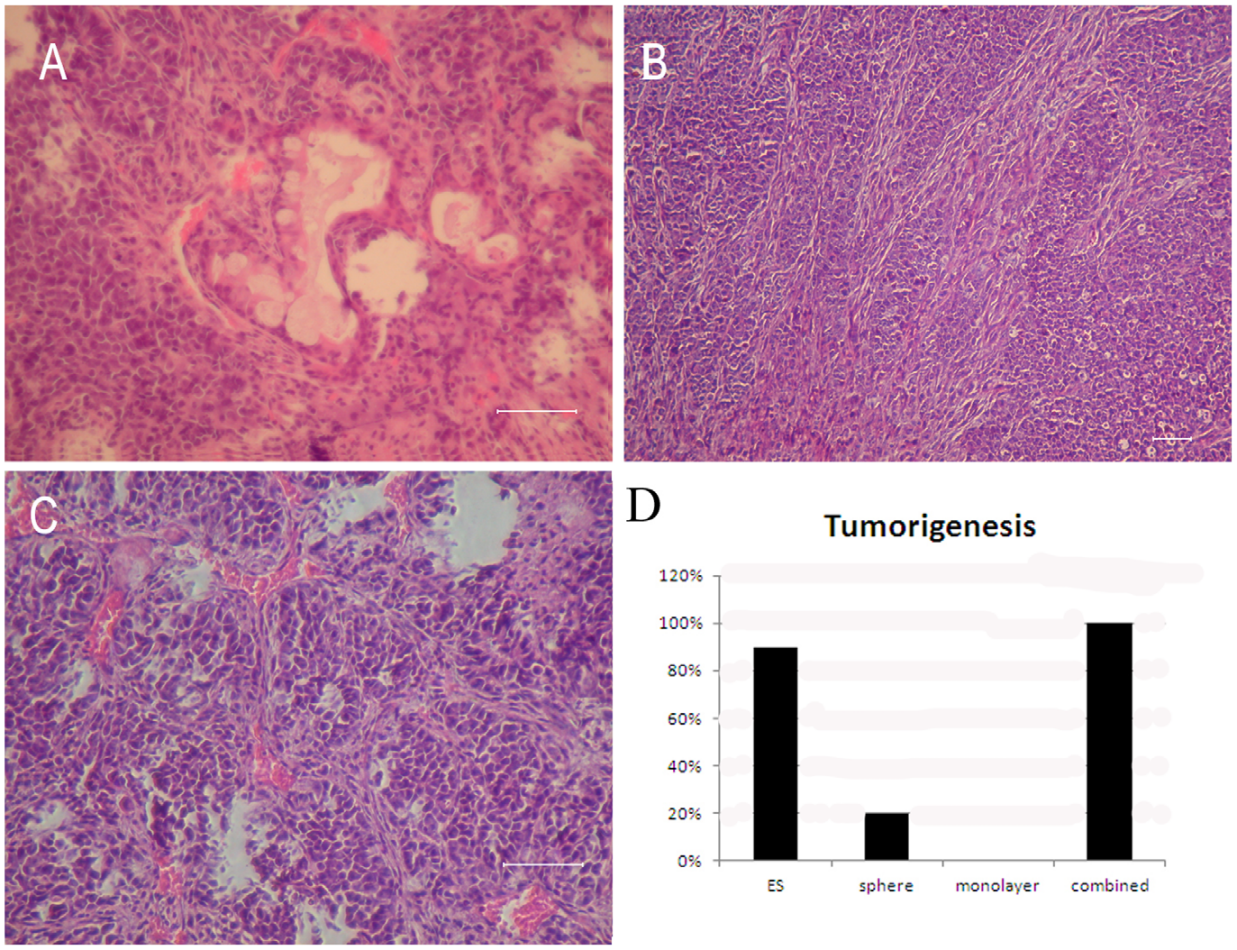
Teratoma formation after transplantation of sphere cells and monolayer cells. Injection of sphere cells and monolayer cells together into immune-deficient mice leads to 100% tumor formation. Sections from excised tumors were stained by H&E for histology. (A) Glandular epithelial (endoderm). (B) Connective tissue (mesoderm). (C) Immature epithelium (ectoderm). (D) When 5×10^6^ cells were used in transplantation, monolayer cells did not form any tumors, while sphere cells formed tumor in 20% of the mice. Transplantation of the sphere cells and monolayer cells together at same quantity of 5×10^6^ led to 100% tumor formation. The same amount of ES cells transplanted turned into tumors in 90% of the animals within 9 weeks. Scale bar, 100 µm.

To test the role of sphere and monolayer cells in tumorigenesis, we increased the cell number for transplantation to 5×10^6^. After transplantation, monolayer cells did not form any tumors while sphere cells formed tumors in 2 out of 10 mice. If sphere and monolayer cells were separated and the same amount (5×10^6^) was transplanted together 3 days later at a ratio of 1∶10, 100% tumor formation was observed. These results suggest that tumor formation in vivo occurs easily when sphere and monolayer cells are together (Fig. 7D). The ES and the stage 4 cells transplanted in same amounts, developed into tumors in 90 and 100% of the animals respectively within 9 weeks.

To demonstrate that the interaction between sphere and monolayer cells is important in tumor formation, we separated the two types of cells and cultured them independently for 1 week. This was followed by transplantation together into nude mice at a ratio of 10∶1. Tumors appeared in all transplanted animals. Tumor formation latency, which is the time between transplantation and appearance of palpable tumor, was 15±3 days when sphere cells and monolayer cells were transplanted together after 7 days in independent culture. This is significantly longer (9±3 days) when the cells were cultured independently for 3 days before transplantation. This indicates that when sphere cells and monolayer cells are cultured separately for longer time before transplantation, more time is needed for them to interact and form tumor after transplantation. Within the neoplasma, tissues of three germ layers were observed including glandular epithelial (endoderm) (Fig. 7A), connective tissue (Fig. 7B) (mesoderm), and immature epithelial (ectoderm) (Fig. 7C).

## Discussion

ESCs have attracted the attention of researchers as potential sources of cell replacement in various diseases. In regenerative research, neural progenitors are needed more often than mature neurons. In previous study, we found that on day 14, stage 4 cells derived from mouse ES can be divided into sphere cells that are neural progenitors, and monolayer cells that are supporting cells playing an important role in accelerating proliferation, reducing apoptosis and prompting differentiation [29].

In this study, we aimed to address the following questions: can stage 4 cells be expanded for as long as 8 weeks and maintain the main characteristics, such as, ability of differentiation into neurons? Do sphere cells and monolayer cells exist during the first 14 days and the subsequent 6 weeks in stage 4 culture? Are sphere cells and monolayer cells in culture neural progenitors and supporting cells respectively? If so, when do the real progenitors in stage 4 become competent to differentiate into neurons and what is the function of cells other than neural progenitors towards neural development?

In our study, using the 5-stage method, stage 4 cells can be expanded for 8 weeks and differentiated into neurons and glial cells. Therefore, stage 4 cells are in general termed as neural-progenitors. Next, we primarily observed that during the first 14 days and the subsequent 6 weeks long culture, stage 4 cells could be divided into two types based on morphology. These two cell types termed sphere cells and monolayer cells differ not only in morphology but also in their ability to differentiate into neurons. Sphere cells are the true neural-progenitors.

Thirdly, cells in stage 4 require 14 days to gain complete ability to give rise to neurons as high as 60–70%. If stage 4 cells as a whole or sphere cells alone started to differentiate into stage 5 before day 14, the percentage of Tuj-1 positive neurons after differentiation reduces significantly. During the following 6 weeks in vitro culture, stage 4 cells require only 5–7 days to form new sphere cells when passaged. These new aggregates, once formed, can give rise to nearly 60–70% Tuj-1 positive neurons after stage 5 differentiation.

Dihné [30] was the first to report the formation of substrate adherent embryonic stem cell induced cell cluster (SENA), which are considered to be neural progenitors after 18 days of culture in stage 4 medium. The difference in stage 4 medium used in his study and ours is the replacement of B27 by N2 in our protocol. Here, sphere cells cultured in stage 4 medium for 14 rather than 18 days were able to differentiate to ∼ 70% neurons. While Dihné’s study did not include the effect of long term culture on the ability of stage 4 cells to give rise to neurons, we found that during the subsequent 6-week culture, the ability of NP cells to give rise to 60∼ 70% neurons did not change. Furthermore, Dihné did not study the function of cells surrounding the neural progenitors. The characteristics of sphere cells in the first 14 days and the subsequent 6 weeks are tightly related to the presence of the surrounding monolayer cells. A novel finding of our study is the supporting role played by monolayer cells in accelerating growth, inhibiting apoptosis and promoting differentiation of neural-progenitors.

Identified by the BrdU incorporation rate, we found that without monolayer cells, the growth of sphere cells was slower during the first 14 days and the subsequent 6 weeks in culture. Similarly, monolayer cells exert an anti-apoptotic effect on the neural progenitors both during the first 14 days and the next 6 weeks long culture period of stage 4. This was exemplified when cells underwent apoptosis in stage 5 differentiation before completing the first 14 days in stage 4.

The anti-apoptotic effect of monolayer cell is important because in cell transplantation research, usually up to 90% of the transplanted cells die within one week [31], [32], and a large proportion of cell death in transplants appears to be apoptotic [33].

Monolayer cells are essential for stage 4 cells to acquire and maintain the ability for neurogenesis. Both during the first 14 days in stage 4 cultures (when sphere cells become competent to give rise to neurons) and the subsequent 6-week long term culture, the presence of monolayer cell results in a higher percentage of differentiated neurons. Although the differentiation protocol we used focused primarily on neurons, the percentage of astrocyte was also higher in the presence of monolayer cells.

Furthermore, to test whether the effect of monolayer cells on sphere cells was unique, we choose mouse fibroblast to replace the monolayer cells. We found that mouse embryonic fibroblasts also had the proliferating and anti-apoptotic abilities of monolayer cells, showing evidence that these two functions are not specific to monolayer cells. However, the effect of monolayer cells on the differentiation of sphere cells to neurons could not be substituted by MEFs. This suggests that the main function of monolayer cells is to promote differentiation of sphere cells into neural lineages.

Cell-cell contact and secreted cytokines were the probable mechanisms by which monolayer cells promoted proliferation of sphere cells and their induction to neurons. The anti-apoptotic function of monolayer cells was mainly due to cell-cell contact.

Based on all of these results, we can make it for sure that the niche, mainly composed of monolayer cells in our experiments, is great helpful in the differentiation of sphere cells into neurons. Stem cell niches are defined as microenvironments that maintain survival, self-renewal, activation, proliferation and regenerative capacity of stem cells. Both in the developing embryo and in vitro, signaling transferred via soluble factors, and or direct cell-cell interactions, contribute to the appropriate regulation of stem cell function [34], [35].

Monolayer cells are also important for tumor formation that occurs after transplantation of sphere cells. The existence of Oct-4 immune-staining during stage 4 is often considered as evidence that the remaining ES cells lead to teratoma formation after transplantation. Since sphere cells contain Oct-4 positive cells, we presumed that tumor formation would occur from sphere cells but not monolayer cells. On the contrary, sphere cells formed tumors only in 2 out of 10 mice while ES and stage 4 cells caused 90 and 100% tumor formation respectively.

In our previous study [29], we tested tumorgenicity of sphere cells and monolayer cells by transplanting10^6^ cells each into nude mice. About 30% of mice that received only 10^6^ sphere cells developed tumors. In contrast, 10^6^ monolayer cells did not result in tumor formation when injected alone into nude mice. However, when both sphere and monolayer cells were injected into nude mice, the rate of tumor formation did not increase. Therefore, we concluded that monolayer cells did not influence tumorgenicity of sphere cells.

In our current study, we increased the number of cells used in transplantation to 5×10^6^ in order to further validate the function of sphere and monolayer cells in tumorigenesis. With higher number of cells transplanted we observed that monolayer cells did not form any tumors while sphere cells formed tumor in 2 out of 10 mice. In contrast, when transplanted together (in quantities of 5×10^6^) at a ratio of 10∶1, sphere and monolayer cells resulted in 100% tumor formation. This suggests that tumor formation in vivo occurs only when monolayer cells and sphere cells are together. In addition, tumor formation latency experiment also confirmed the importance of the interaction between monolayer cells and sphere cells in tumor formation.

Our results showed that the separation of monolayer cells and sphere cells is a good model to study the interaction of NPCs and their niche. To study the interaction of NPC and its niche is good to find more soluble factors and transcript factors useful in increasing the percentage of specific neurons in differentiation. Some research made breakthrough recently. For example, Vazin et al. found PA6 cell surface activity is required for neural differentiation of hESC, but secreted factors are required for the specific DA neuron-inducing effect. Using more comprehensive array studies, they have identified a large number of candidate molecules potentially responsible for the SDIA effect [25]. Swistowska showed that medium conditioned by the stromal cell line PA6 (PA6-CM) can induce dopaminergic differentiation in neural stem cells (NSCs) derived from hESCs but not directly from hESCs, indicating that soluble factors produced by PA6 cells act at the NSC stage to specify a dopaminergic fate. They also found Shh and FGF8 can substitute for PA6-CM at the NSC induction stage. Shh is indeed one of the active agents in PA6-CM and is likely an important soluble dopaminergic inducing factor secreted by stromal cells and acts after the neural fate determination [36].

In summary, stage 4 culture creates two distinct cell populations that influence each other. Sphere cells are committed to form neural cells and monolayer cells are the supporting cells that prompt proliferation, decrease apoptosis and enhance differentiation of sphere cells. Significantly, we found the importance of the interaction between the two cell types in neurogenesis and tumorigenesis.

Further work is needed in particular to determine the function of monolayer cells on development of functional neurons, such as tyrosine hydroxylase (TH) positive neurons, GABA neurons, etc. In addition, sphere cells may also be a heterogeneous group of cells and it is yet to be determined which cell types and genotyping markers are more committed to become neural precursors or neurons. Although sphere cells can differentiate into more neurons with the help of monolayer cells in vitro, it is not known if the same is true in vivo.

## Materials and Methods

### Ethics Statement

All animal protocols used in the present study were reviewed and approved by the Ethics Committee on Laboratory Animal Care at Capital Medical University.

### 1. ES Cell Culture and Differentiation by the 5-stage Method

We used the mES line R1, passed 30–50 times, in our study. The usage of ES cells and laboratory animals was approved by the Ethical Committee of Xuanwu Hospital at Capital Medical University, Beijing.

Mouse ESCs were cultured in knockout Dulbecco’s modified eagle’s medium (DMEM; Invitrogen, Carlsbad, CA, USA) supplemented with 15% serum (Invitrogen), 2 mM GlutaMax (Invitrogen), 1% non-essential amino acids (Cambrex Bio Science, New Jersey, NJ, USA), 50 U/mL penicillin/streptomycin (Invitrogen), 0.1 mm β-mercaptoethanol (Invitrogen, Minneapolis, MN, USA) on STO feeder cell layer (ATCC, USA). The cultures were free of mycoplasma throughout the experiment. Karyotype of the ES cells was normal, 46XX, as analyzed in passages 30 and 50. ES cells were termed stage 1 in the 5-stage differentiation method.

For neural differentiation, the mESC colonies were dissected with the use of 0.25% trypsin-EDTA into single cells and plated on to gelatin coated dishes and cultured for three passages. The cells were then transferred to bacterial petri dishes to form embryonic bodies in the mES medium without leukemia inhibitory factor, LIF. The embryonic bodies were termed stage 2. Four days later, only the EBs in suspension were transferred into gelatin coated tissue culture dish and the selection of neural progenitors was initiated by replacing the medium with ITSFn (insulin, transferrin, selenium and fibronectin). The cells in ITSFn medium were termed stage 3 that lasted for 10 days.

The cell clusters formed in stage 3 were then disassociated by using 0.25% trypsin-EDTA and plated at the density of 1×10^5^/cm^2^ and cultured for 14 days in DMEM/F-12 (Invitrogen) supplemented with N2 (Invitrogen), 2 mM GlutaMax (Invitrogen) and 10 ng/mL basic fibroblast growth factor, bFGF (R&D systems) in 6-well plates pre-coated with 10 µg/mL polyornithine (Sigma, St. Louis, http://www.sigmaaldrich.com). The cells in the medium with bFGFwere termed stage 4. In this study, the mouse stage 4 cells, containing spheres and monolayer cells, were passed once every 5–7 days and the medium was changed every day. Each time after passing, the stage 4 cells were induced into stage 5 differentiation to test their ability to giving rise to neurons.

### 2. Separation of the Cell Types in Stage 4 during the First 14 Days and the Subsequent 6 Weeks

In our study, we divided stage 4 into the first 14 days, and the subsequent 6 weeks of continuous culture. The monolayer cells which adhered to the dish and cell aggregates that were scattered over the monolayer cells formed on day 2–3 in stage 4. To obtain these pure sphere cells, stage 4 cells were digested with 0.3 µg/ml collagenase IV (Sigma-Aldrich, St. Louis, http://www.sigmaaldrich.com) for 5–8 minutes at 37°C. During the collagenase treatment, dishes were gently shaken to detach the spheres from the monolayer cells.

### 3. Identification of Neural Progenitors

To identify whether neural progenitors reside in sphere cells or the monolayer cells in the first 14 days and the subsequent 6 weeks culture, the sphere cells were separated at different time points. The spheres and monolayer cells were then differentiated in stage 5 medium for 7 days before staining with neural and glial lineage antibodies.

### 4. The Maturation of NPCs in Stage 4

Previous studies have described the duration of stage 4 as 7 days [8]–[10]. In our experiment, we wanted to determine when the sphere cells get the full potential to form neurons. We started differentiation of stage 5 on days 3, 7, 10, and 14 in stage 4. Longer time points were not chosen because the cells cultured in stage 4 are nearly 90–100% confluent by day 14, and if not passed, many cells would die. After the final differentiation to stage 5, the percentage of neurons and astrocytes were quantified by cell counting.

### 5. Immunocytochemical Characterization of Differentiated Cells

Cells after 5 stage differentiation were fixed in 4% paraformaldehyde (PFA) at room temperature for immunocytochemical analysis. After fixation, cells were treated with 0.1% Triton X-100 and 1% bovine serum albumin (BSA) for 30 min and then blocked with 10% normal goat serum in 0.1% phosphate-buffered saline (PBS). Cells were incubated overnight in PBS with primary antibodies, 0.1% Triton X-100 and 1% BSA at 4°C. The next day, cells were washed with 0.1% PBS and incubated in the same solution with secondary antibodies (1∶400). Finally, cells were washed with PBS and counterstained with 4′6-diamidino-2-phenylindole (DAPI; Vector Laboratories, Peterborough, UK).

The antibodies included polyclonal goat anti- mouse glial fibrillary acidic protein (GFAP; 1∶600, AF2594; R&D Systems) and monoclonal mouse anti-mouse Tuj-1 (1∶400, MAB1637, Millipore, USA). The secondary antibodies were Cy2 or Texas red conjugated goat anti-mouse or goat anti-rabbit IgG (Immuno-Jackson, Baltimore, PA, USA).

To exclude the false absorption of secondary fluorescent antibody by cell aggregates, we accurately measured the cell numbers in the spheres. The spheres were gently dissociated by accutase to avoid cell death. All cells were centrifuged, re-suspended in PBS, plated in 2% gelatin and 0.05% chromic potassium sulfate coated slides and fixed with 4% PFA before immuno-histochemistry staining was performed.

### 6. Cell Counting

Percentage of neurons or astrocytes was determined by counting the numbers of Tuj-1 positive or GFAP positive cells and the numbers of DAPI stained nuclei using a fluorescent microscope at 20× magnification (TE2000U, Nikon, Japan). Ten visual fields were randomly selected and counted.

### 7. Teratoma Formation

We separated the sphere cells and monolayer cells and injected them into nude mice to find their respective roles in tumor formation. To study their interaction, the cells were initially cultured for 1 week independently followed by combining them at a 10∶1 ratio (sphere cells: monolayer cells) before infusing into the right flank of nude mice.

Sixty male BALB/c-nude mice (Laboratory of Animal Science Institute of Chinese Academy of Medical Sciences, Beijing, http://www.cnilas.org/html/en/) were used in the teratoma formation test. Mice were kept in a specific pathogen-free (SPF) unit at the Capital Medical University. All experiments were performed after approval of the Committee for Animal Care and Use of the Faculty of Sciences at the Capital Medical University.

The animals were divided into 6 groups and injected with (1) sphere cells, (2) monolayer cells, (3) sphere cells+monolayer cells immediately after combining, (4) sphere cells+monolayer cells one week after combining, (5) ES cells and (6) stage 4 cells. For each animal, 5×10^6^ cells were suspended in 100 µL PBS and injected into the right flank [20]. Tumor latency was monitored over a period of 90 days. At the end of the observation period, mice were sacrificed.

### 8. Effect of Monolayer Cells on Sphere Cells

In this section, we compared the proliferation, apoptosis, and neurogenesis of stage 4 cells as a whole and sphere cells alone. To avoid the impact of collagenase disassociation on the characteristics of sphere cells and monolayer cells, the stage 4 cells are actually “co-culture” cells, which was a combination of sphere and monolayer cells after collagenase disassociation. To prepare the “co-culture” cells, the two cell types in stage 4 were separated at first, then after counting, we concluded that the quantitative ratio of sphere cells to monolayer cells is approximately 10∶1. We first plated 2×10^4^/well monolayer cells in each well of the 6-well plate, followed by 2×10^5^ sphere cells onto the monolayer cells. For the combined cells to setup crosstalk again, the “co-culture” cells were kept in stage 4 medium for 2 days before further experiments were performed.

To analyze whether the effect of proliferation, apoptosis and differentiation on sphere cells was exclusive to the monolayer cells, we selected week 5 as the time point for examination and another monolayer feeder cells, the CF1 mouse embryonic fibroblasts (MEFs) (Shanghai Institute of Biochemistry and Cell biology, http://www.sibcb.ac.cn/eindex.asp), as the substitution of monolayer cells. To exclude the influence of MEF or monolayer cell growth on the characteristics of sphere cells, the MEFs or monolayer cells were treated with 10 mg/ml mitomycin-C for 150 min (Sigma, St. Louis, MO) and then washed three times with fresh medium. The MEFs or monolayer cells were then permitted to recover overnight before sphere cells were seeded.

To study the effect of cell-cell contact and secreted factors on the proliferation, apoptosis and differentiation of sphere cells, sphere cells at week 5 were divided into 5 groups; one group was cultured with monolayer cells in stage 4 medium, the second with a conditioned medium from monolayer cells, the third only with stage 4 medium, the fourth with a conditioned medium from MEFs, and the fifth with MEFs in stage 4 medium.

#### 8.1 The effect of monolayer cells on the proliferation of sphere cell

The time points of day 7, 10, and week 5, 8 in stage 4 were selected to study the effect of monolayer cells on the proliferation of sphere cell. Cells used for the combined culture were brought together after separation. The separated and re-combined cells were then cultured for 2 days. During the last 15 hours of the co-culture, 10 µM 5-bromo-2-deoxyuridine (BrdU) was used to identify the number of cells still in proliferation. Cells were fixed in 4% PFA and immune-histological staining was performed to test the percentage of BrdU positive cells.

#### 8.2 The effect of monolayer cells on the neurogenesis of sphere cells

Day 3, 7, 10, 14, and weeks 5 and 8 in stage 4 were selected to study the effect of monolayer cells on the neurogenesis of sphere cells. The cells were transferred into stage 5 medium for 7 days differentiation. We further selected week 5 to study the effect of cell-cell contact and secreted cytokines on the neurogenesis of sphere cells, and whether the effect of monolayer cells can be replaced by MEFs.

#### 8.3 Effect of monolayer cells on apoptosis of the sphere cells

We separated sphere cells on days 3, 7, 10, 14 and weeks 5 and 8. Cells used for the combined culture were brought together after separation. Two days later all cells were stained by active caspase 3 antibody and positive cells were counted. The results were confirmed by western blot. For analysis of protein levels, cells were rinsed twice with ice-cold PBS, lysed with ice-cold lysis buffer and were centrifuged at 14,000 g for 10 minutes at 4°C; the supernatant was then mixed with SDS sample buffer, boiled and separated through 12% SDS-PAGE gels. After electrophoresis and being transferred to nylon membranes by electrophoretic transfer, the proteins were blocked and incubated overnight at 4°C with primary antibody and 2 hours with horseradish peroxidase-conjugated secondary antibodies. Bands were visualized by enhanced chemoluminescence.

Besides active caspase 3 staining, the “FITC Annexin V Apoptosis Detection Kit I ” (BD Pharmingen, http://www.bdbiosciences.com/) was also be used to study the effect of cell-cell contact and secreted cytokines on the apoptosis of sphere cells, and whether the effect of monolayer cells can be replaced by MEFs.

The apoptosis were analyzed according to the manufacturer’s instructions. In brief, cells were rinsed with ice-cold PBS and resuspended in binding buffer at a concentration of 1×10^6^ cells/ml. Approximately 1×10^5^ cells was transferred to a 5 ml culture tube, then 5 uL FITC-Annexin V and 5 uL propidine iodide (PI) was added to the cells and incubated for 15 minutes at room temperature in the dark. The cells were immediately analyzed on a FACSC-LSR equipped with CellQuest software(BD, http://www.bd.com).

### 9. Statistical Analysis

Microsoft Excel (Microsoft, http://www.microsoft.com) software was used for calculating mean and SD, and constructing histogram plots. Samples that passed the normal distribution test were subjected to t-test. The t-tests were used to establish null hypothesis between any two groups based on unpaired, unequal variance and two-sided model. Statistical significance was considered if p value was <0.05.

## Supporting Information

Figure S1 **Sphere cells began to differentiate on days 3, 7, 10 and 14 in stage 4 with or without monolayer cells.** In the later 3 time points, astrocyte percentage in the presence of monolayer cells were more than in their absence (p<0.05). Green,GFAP; Blue, DAPI. Scale bar, 20 µm.
(TIF)
