# Supplementary figures and images for: Function of Mouse Embryonic Stem Cell-Derived Supporting Cells in Neural Progenitor Cell Maturation and Long Term Cxpansion
Source: PLoS One. 2013 Jan 14;8(1):e54332. doi: 10.1371/journal.pone.0054332 (PMC3544757; doi:10.1371/journal.pone.0054332)

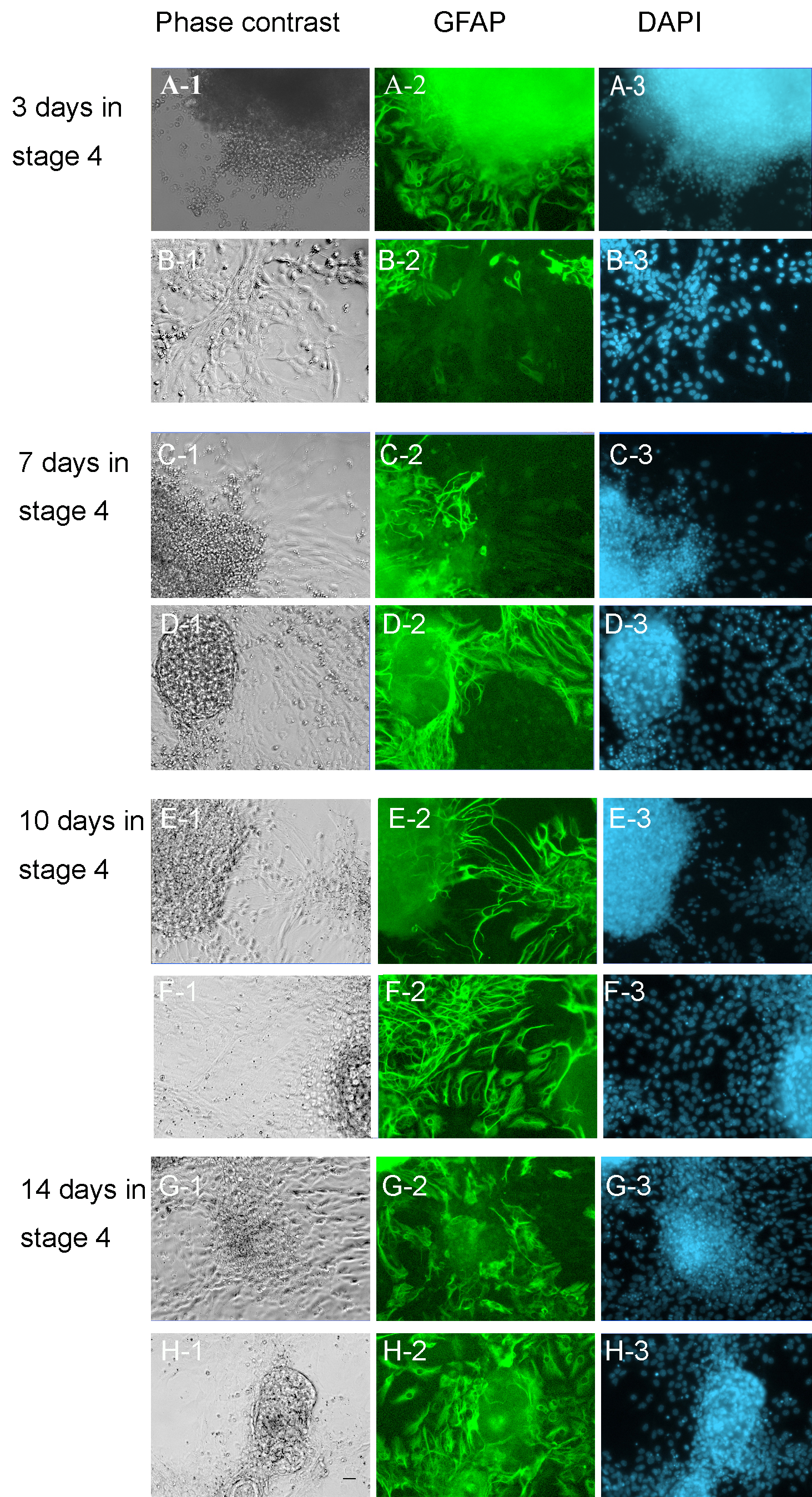

Supplement: Figure S1 — Sphere cells began to differentiate on days 3, 7, 10 and 14 in stage 4 with or without monolayer cells. In the later 3 time points, astrocyte percentage in the presence of monolayer cells were more than in their absence (p<0.05). Green,GFAP; Blue, DAPI. Scale bar, 20 µm. (TIF) [file pone.0054332.s001.tif]
